# Sleep Disturbances and Suicidality in Posttraumatic Stress Disorder: An Overview of the Literature

**DOI:** 10.3389/fpsyt.2020.00167

**Published:** 2020-03-10

**Authors:** Franziska C. Weber, Christine Norra, Thomas C. Wetter

**Affiliations:** ^1^Department of Psychiatry and Psychotherapy, University of Regensburg, Regensburg, Germany; ^2^LWL Hospital Paderborn, Psychiatry-Psychotherapy-Psychosomatics, Ruhr University of Bochum, Bochum, Germany

**Keywords:** sleep, sleep disorders, suicidality, PTSD (post-traumatic stress disorder), insomnia, nightmares

## Abstract

A causal relationship between sleep disturbances and suicidal behavior has been previously reported. Insomnia and nightmares are considered as hallmarks of posttraumatic stress disorder (PTSD). In addition, patients with PTSD have an increased risk for suicidality. The present article gives an overview about the existing literature on the relationship between sleep disturbances and suicidality in the context of PTSD. It aims to demonstrate that diagnosing and treating sleep problems as still underestimated target symptoms may provide preventive strategies with respect to suicidality. However, heterogeneous study designs, different samples and diverse outcome parameters hinder a direct comparison of studies and a causal relationship cannot be shown. More research is necessary to clarify this complex relationship and to tackle the value of treatment of sleep disturbances for suicide prevention in PTSD.

## Introduction

Suicide is a leading cause of death worldwide with at least 788,000 annual deaths from suicide around the world in 2015 according to WHO data (1). Sleep disturbances have been shown to be a risk factor for mental disorders for decades, and are an independent risk factor for suicidal behavior (2). However, not only sleep problems in general have evolved as an evidence-based risk factor for suicide (3, 4), but short sleep duration also seems to be associated with suicidality (5, 6). Moreover, insomnia has also been proposed to be an independent risk factor for suicidality (7, 8). However, the association is weaker after controlling for depression (9, 10). Further, there is also evidence for an association between nightmare disorder and suicidality (11–13). However, the specific contributions of nightmare disorder to suicidality remain unclear (14). Unfortunately, nightmares are under-reported, therefore detection and treatment are often insufficient (15).

### PTSD and Sleep Disturbances

The lifetime prevalence of posttraumatic stress disorder (PTSD) is estimated to be about 8.0% in the general population (16, 17), and sleep disturbances are considered as a core feature of PTSD (18). There is a high prevalence of nightmares (up to 70%) in patients with PTSD (19), and the rate of sleep disturbances in PTSD is estimated to be in the same range as that of nightmares and insomnia at about 40% (20). A bidirectional relationship between PTSD and sleep disturbances has been purported (21, 22). This is in line with the hypothesis that it is biologically plausible for sleep disturbance to be mechanistically transdiagnostic, suggesting that sleep disturbances are related to the onset and course of several psychiatric disorders. Specifically, increased activity of the noradrenergic system during rapid-eye-movement-(REM)-sleep, REM-sleep fragmentation and reduced activity of the medial prefrontal cortex during REM-sleep may play a pivotal role with respect to sleep fragmentation and emotion dysregulation in PTSD (23).

Apart from sleep disturbances and nightmares being an integral diagnostic criteria of PTSD, specific sleep disorders such as obstructive sleep apnea syndrome (OSAS), REM-sleep behavior disorder, and periodic limb movement disorder have been reported to be disproportionately frequent in PTSD patients (24). Several studies have shown a high prevalence of OSAS in PTSD patients, and it is suggested that OSAS plays a role in the pathophysiology of PTSD symptoms (25–27). There is also evidence for a positive correlation between effective OSAS treatment and reduced PTSD severity (28, 29). Sleep terrors, nocturnal panic attacks, and simple and complex motor behaviors including vocalizations and acting out dreams may also occur during sleep in patients with PTSD (23). These features were recently described as “Trauma Associated Sleep Disorder” which may also occur without the presence of other typical PTSD symptoms (30, 31).

### PTSD and Suicidality

Numerous studies have shown that PTSD represents a risk factor for suicidal thoughts and behaviors (32, 33). In addition, there is evidence for a mediating role of depression in the association of PTSD and suicidality (34). Comorbid depression may amplify the suicide risk synergistically beyond an additive effect (35). Indeed, there is evidence of an increased risk of death by suicide in PTSD patients in general (36, 37) even compared to trauma-exposed people (38, 39). Considering suicide risk, it is noteworthy that PTSD is one of the few psychiatric disorders that distinguish those who think about suicide from those who make suicide attempts (40). A meta-analysis has shown that PTSD is reliably more common in attempters than ideators, but these effects seem to be modest rather than strong (41).

### PTSD, Sleep Disturbances and Suicidality

There are only few studies reporting on the relationship between PTSD, sleep disturbances, and suicidality. A meta-analysis of sleep disturbances and suicidal thoughts and behaviors including studies on mixed samples (42) identified only one study on their relationship to PTSD (43); another meta-analysis and systematic review about the association between sleep disturbances and suicidal behaviors in patients with psychiatric diagnoses (44) also found the same study by Krakow et al. (43). Moreover, a systematic review of the relationship between sleep disturbance, suicidal ideation, suicide attempts, and suicide among adults (45) provided three studies for PTSD (46–48) and seven studies for veterans in consideration of a possible PTSD diagnosis (49–55).

## Literature Search and Results

We conducted a comprehensive search of MEDLINE/PubMed databases using Medical Subject Headings terms in various combinations to identify studies that examined aspects of sleep disorders, suicidality and PTSD. Furthermore, we identified several studies by searching through references of identified studies, reviews and meta-analyses. In total, we identified 30 studies (Tables 1–3).

**Table 1:** Studies referring to PTSD, suicidality and sleep disturbances (insomnia).

**References**	**Core issue**	**Sample**	**Sex (male)**	**Measurements**	**Main findings**
*Betts et al. (46)*	Association between the risk of SI in individuals with PTSD symptoms on comorbid sleep disturbance	Participants of the MUSP (*n* = 2,465) with PTSD-symptoms: 9.4%; proportion of those with PTSD-diagnosis not mentioned	48.0%	Single item (SI), shortened version PSQI (sleep quality), CIDI-Auto (PTSD)	- PTSD symptoms did not directly predict SI when adjusting for MDD symptoms, polyvictimization, and gender - PTSD symptoms had an indirect effect on SI via past-month sleep disturbance
Bishop et al. (49)	Association of sleep disturbance with SI after controlling for age, alcohol dependence, depression, and PTSD	Veterans (*n* = 654), with PTSD: 24.3%	95,3%	PSS (SI), single item PCL (sleep disturbance), PTSD (PCL)	Sleep disturbance was a predictor of SI, even after controlling for age, alcohol dependence, PTSD, and depression
Bishop et al. (56)	Association among sleep disorders and SA after controlling for several mental disorders, medical comorbidity, and obesity	Veterans (data base review) (*n* = 60,102, 1:1 case control with no SA), with PTSD: 24.7%	87.1%	Data extraction for SA, sleep disturbance, mental disorders	- Insomnia was associated with SA - Sleep medicine visits were associated with a reduced risk of SA in sleep disordered patients
Britton et al. (57)	Associations among insomnia symptoms, PTSD symptoms and depressive symptoms, IPT variables, and risk for SB	Veterans (*n* = 392), with PTSD: 41.8%	69.6%	SBQ-R (SB), ISI (insomnia), PCL-M (PTSD)	- Insomnia symptoms may be associated with increased PTSD and depressive symptoms - PTSD emerged as a mediator between insomnia and SI
Bryan et al. (50)	Identifying clinical variables (incl. PTSD) associated with suicidality in military personnel with mTBI	Deployed soldiers (*n* = 158), with mTBI: 85.4%; proportion of PTSD not mentioned	93%	SBQ-R (suicidality), ISI (insomnia), PCL-M (PTSD)	Suicidality was significantly associated with depression and the interaction of depression with PTSD symptoms
Bryan et al. (58)^*^	Associations of insomnia severity with SI, and SB	3 samples of active duty military (*n* = 380); proportion of PTSD not mentioned	76.6%	BSSI (SI), ISI (insomnia), PCL (PTSD)	Association between sleep disturbances and SI (concurrent/prospective) in all samples; this effect was no longer present after adjusting for age, gender, depression, and PTSD
Chakravorty et al. (51)	Association between SI and insomnia symptoms adjusted for socio-demographic, psychiatric and addiction-related variables	Outpatient veterans, misusing alcohol (*n* = 162), with PTSD: 27%	93%	PSI (SI, SB, SA), single item PSQI (sleep quality), PCL (PTSD)	- After controlling for psychopathology, a significant association between insomnia and SI was no longer present; poor sleep quality remained associated with increased SI - SI was not associated with sleep duration
Davidson et al. (48)	Association between exercise and suicide risk including potential mediators (i.e., sleep disturbance, PTSD symptoms, depression)	Veterans admitted to a residential rehabilitation program for PTSD (*n* = 346)	81%	8 items of BDI II(suicidality), PSQI (sleep quality), PCL-M without sleep item (PTSD)	- Direct negative association between suicide risk and exercise, indirectly through association with depressive symptoms and sleep quality - PTSD symptoms did not mediate the relation between exercise and suicide risk
*Dell'Osso et al. (47)*	Impact of alterations in rhythmicity and vegetative function (incl. sleep disturbances) as correlates of suicidality	Civilian inpatients/outpatients with PTSD (*n* = 65); patients with current (*n* = 20) or former depression (*n* = 14) were excluded	50.8%	MOOD-SR (SI/SA/sleep disturbances)	All MOODS-SR sub-domains (rhythmicity, sleep, appetite/weight, physical symptoms) were associated with an increased likelihood of SI; changes in appetite/weight were associated with greater OR of SA
Don Richardson et al. (52)	Association between SI and sleep after controlling for probable PTSD, MDD, GAD, AUD	Veterans (*n* = 324)/active duty military (*n* = 80), with PTSD: 72.8%	92.4%	Single item PHQ-9 (SI), quantitative single item (sleep disturbances resp. nightmares), PCL-M (PTSD)	- Sleep disturbances did not predict SI - Probable MDD emerged as a significant predictor
Don Richardson et al. (59)	Mediating role of depression in the relationship between: 1) sleep disturbances and SI, and 2) trauma-related nightmares and SI after controlling for PTSD-, anxiety- and alcohol-use-severity	CAF personnel (17.6%)/veterans (82.4%), (*n* = 663), with PTSD: 72.6%	91%	Single item PHQ-9 (SI), quantitative single item (sleep disturbances resp. nightmares), PCL-M (PTSD)	Sleep disturbances were associated with SI as a function of depressive symptoms
Fisher et al. (60)	The moderating role of agitation within the relationship between insomnia and current SI	U.S. military personnel (*n* = 937); proportion of PTSD is not mentioned	75.3%	BSSI/DSI-SS (SI), ISI (insomnia), PCL-M (PTSD)	- Significant association between insomnia and SI only at high levels of agitation - PTSD symptoms, depressive symptoms, and lifetime number of SA were each associated with greater levels of agitation, insomnia, and current SI
Kachadourian et al. (61)	Association between individual symptoms of PTSD and measures of functioning, quality of life, and SI	U. S. military veterans (*n* = 1,484), with PTSD: 10.9%, trauma-exposed: 85,4%	89.8%	Single item on the PHQ-9 (SI), PCL-5 (sleep difficulties/ nightmares/PTSD)	- Sleep difficulties explained problems in physical functioning/quality of life after adjustment for severity of PTSD/depressive symptoms - No association between SI and sleep difficulties
Kim et al. (62)	The mediating role of AUD and insomnia in the relationship between PTSD symptoms and SI	Korean firefighters (*n* = 7,190), with PTSD: 3.6%	90%	PHQ-9 suicide item (SI), AIS (insomnia), PCL (PTSD)	AUD and insomnia mediated the relationship between PTSD symptoms and SI
Luxton et al. (63)	Prevalence and impact of short sleep duration in redeployed OIF soldiers	Redeployed OIF soldiers (*n* = 2,738), with PTSD: 15.4%	96%	HRA II (SA), 2 items (sleep duration/sleep quality), PC-PTSD (PTSD)	- SSD was a significant predictor of suicide risk - SSD was the strongest predictor of PTSD
McClure et al. (64)	To determine the prevalence of factors that may serve as warnings of acute suicidality risk	Veterans attending an urgent care psychiatric clinic (*n* = 473), with PTSD: 49%	89%	SWS survey (SI/insomnia/ hypersomnia), PC-PTSD (PTSD)	- Past week SI and sleep disturbances were among others a highly prevalent warning sign - 97% endorsed at least one warning sign, participants with MDD and/or PTSD endorsed the largest number of warning signs
Morgan et al. (65)	The relationship between sleep issues, mental health (perceived stress, PTSD symptoms, and depressive symptoms), and SI	Military service members (*n* = 891), with PTSD: 13%	95.5%	Single item (SI), PROMIS (sleep disturbances), PCL-C (PTSD)	PTSD, perceived stress, and depressive symptoms mediated the relationship between sleep issues and SI; after accounting for mental health symptoms, sleep no longer had a direct effect on SI
Pigeon et al. (53)^*^	Role of sleep disturbance in time to suicide since the last treatment visit among veterans receiving VHA services	Suicide decedents (*n* = 423) Visit of the VHA (*n* = 381); proportion of PTSD is not mentioned	99.7%	Chart review for number of days between last visit and death, sleep disturbances, and psychiatric symptoms	Veterans with sleep disturbance died sooner after their last visit than did those without sleep disturbance, after adjusting for the presence of mental health or substance use symptoms, age, and region
Pigeon et al. (66)^*^	bCBTi delivered to veterans endorsing SI with a diagnosis of MDD and/or PTSD	Veterans (*n* = 54, RCT 1:1 TAU vs. TAU plus bCBTi); proportion of PTSD not mentioned	80%	C-SSRS (SI), ISI (insomnia), PCL-M (PTSD)	- No significant effect of bCBTi on SI intensity - Effects were large on insomnia and depression with no effect on PTSD
Ribeiro et al. (54)^*^	Relationship between insomnia symptoms and SI/SB after controlling for depressive symptom severity, hopelessness, PTSD diagnosis, anxiety symptoms, drug and alcohol abuse	Military personnel (*n* = 311), with PTSD: about 20%	82%	MSSI (SI), insomnia symptom index (sleeplessness), diagnosis (PTSD)	- Insomnia symptoms were cross-sectionally associated with SI - Insomnia symptoms were unique predictors of SA longitudinally after controlling for baseline self-insomnia resp. depressive symptoms and hopelessness
Ribeiro et al. (67)	Association between PTSD status and functional impairment (sleep quality, alcohol use, social problem-solving, work and social adjustment) among suicidal military inpatients	Suicidal military psychiatric inpatients and a lifetime history of at least one SA (*n* = 166), with PTSD: 38%	65%	C-SSRS (SI/SB), PSQI (sleep quality), MINI (PTSD)	- Patients with PTSD reported disturbed sleep and reduced social and work adjustment, association was no longer significant after adjusting for gender and psychiatric comorbidity - Those with a greater number of psychiatric comorbidities demonstrated higher likelihood of meeting PTSD criteria
Richardson et al. (68)	The relationship between insomnia, SI, and past-year mental health status	Canadian Regular Forces personnel (*n* = 6,700), with PTSD: 5.3%	86.1%	Single item (past-year SI/insomnia), WHO-CIDI (PTSD)	- Both insomnia and number of mental health conditions incrementally increased the risk of SI - Insomnia significantly increased the odds of SI, but only among individuals with no or one mental health condition
*Selaman et al. (69)*	To determine specific DSM-IV symptoms of PTSD that are independently associated with SA	Data from wave 2 of the NESARC (*n* = 34,653), with PTSD: *n* = 2,322	27.9%	Single item (nightmares/sleep disturbances/SA), DSM-IV-criteria (PTSD)	- Increasing numbers of re-experiencing and avoidance symptoms were correlated with SA - No association between SA and sleep disturbances (A)OR about 0,6
Swinkels et al. (55)	Association of sleep duration and sleep quality with mental health and SI	U.S. Afghanistan/Iraq era veterans (*n* = 1,640), with PTSD: 31%	80%	BSSI (SI), PSQI-A (sleep quality/ duration), SCIDI/P (PTSD)	- Very SSD (≤ 5 h of sleep) and LSD (≥ 9 h) were each (after adjusting for diverse covariates) associated with increased odds of current PTSD, MDD, and smoking - Poor sleep quality was associated with PTSD, PD, MDD, SI, and risky drinking
Wang et al. (70)^*^	Association of pre-deployment insomnia with post-deployment PTSD and SI	U. S. Army soldiers (*n* = 8,558, cross-sectional, *n* = 4,645, longitudinal), with PTSD 11.9%	94.7%	C-SSRS (suicidality), items of the Brief Insomnia Questionnaire (insomnia), PCL (PTSD)	Pre-deployment insomnia was associated with increased risk of post-deployment PTSD and SI even after adjusting for socio-demographic characteristics and prior deployment history

* *Denotes studies with longitudinal designs; all others are cross-sectional studies*.

*Cursive references denotes studies on civilian samples*.

**Table 2:** Studies referring to PTSD, suicidality, and nightmares.

**References**	**Core issue**	**Sample**	**Sex (male)**	**Measurements**	**Main findings**
Bishop et al. (56)	Association among sleep disorders and SA after controlling for several mental disorders, medical comorbidity, and obesity	Veterans (data base review) (*n* = 60,102, 1:1 case control with no SA), with PTSD: 24.7%	87.1%	Data extraction for SA, sleep disturbance, mental disorders	- Nightmares were after controlling for psychiatric disorders no longer associated with SA - Sleep medicine visits were associated with a reduced risk of SA in sleep disordered patients
Don Richardson et al. (52)	Association between SI and sleep after controlling for probable PTSD, MDD, GAD, AUD	Veterans (*n* = 324)/Active duty military (*n* = 80), with PTSD: 72.8%	92.4%	Single item PHQ-9 (SI), quantitative single item (sleep disturbances resp. nightmares), PCL-M (PTSD)	- Nightmares did not predict SI - Probable MDD emerged as the most significant predictor
Don Richardson et al. (59)	Mediating role of depression in the relationship between: (1) sleep disturbances and SI, and (2) trauma-related nightmares and SI after controlling for PTSD-, anxiety-symptom-, and alcohol-use-severity	CAF personnel (17.6%)/ veterans (82.4%) (*n* = 663), with PTSD: 72.6%	91%	Single item PHQ-9 (SI), - quantitative single item (sleep disturbances resp. nightmares), PCL-M (PTSD)	Trauma-related nightmares were associated with SI as a function of depressive symptoms
*Littlewood et al. (71)*	Mechanism of the relationship between nightmares and SB in consideration of perceptions of defeat, entrapment, and hopelessness	Trauma-exposed patients (*n* = 91) with PTSD-symptoms, with confirmed PTSD: *n* = 51, history of PTSD diagnosis: *n* = 36	26%	SBQ-R (suicidality), in each cases 2 items of CAPS (nightmares/insomnia), CAPS (PTSD)	- SB were higher in participants who experienced nightmares - Nightmares were directly or indirectly associated with SB, through perceptions of defeat, entrapment, and hopelessness, independent of comorbid insomnia and depression.
McCall et al. (72)^*^	Examining whether treatment of nightmares with prazosin (nighttime-only) would reduce SI in suicidal PTSD patients	20 adult, suicidal PTSD patients with nightmares in a RCT over 8 weeks; *n* = 2 were militarian	15%	SSI (SI), DDSNSI (nightmares), ISI (insomnia), CAPS (PTSD)	- All psychometric measures improved over 8 weeks - Nighttime measures of nightmares and insomnia showed less improvement in the prazosin group - No significant changes in daytime measures of SI and daytime-only PTSD symptoms
Raskind et al. (73)^*^	RCT of Prazosin for PTSD for 26 weeks with three primary outcome measures	Veterans with chronic PTSD and frequent nightmares (*n* = 304, 1:1 Placebo/Prazosin)	97.7%	Adverse event (SI), CAPS (nightmares), PSQI (sleep quality), PCL-M (PTSD),	- Prazosin did not improve distressing dreams or sleep quality - Adverse event of new or worsening SI occurred in 8% of participants with prazosin vs. 15% with placebo
*Selaman et al. (69)*	To determine specific DSM-IV symptoms of PTSD that are independently associated with SA	Data from wave 2 of the NESARC (*n* = 34,653), with PTSD: *n* = 2,322	27.9%	Single item (nightmares/sleep disturbances/SA), DSM-IV criteria (PTSD)	Association between nightmares and SA, this effect disappeared after adjusting for covariables

* *Denotes studies with longitudinal designs; all others are cross-sectional studies*.

*Cursive references denotes studies on civilian samples*.

**Table 3:** Studies referring to PTSD, suicidality, and sleep-related breathing disorders.

**Reference**	**Core issue**	**Sample**	**Sex (male)**	**Measurements**	**Main findings**
*Gupta and Jarosz (80)*	Diagnosing OSAS by sleep study and SI in patients with PTSD	Civilians with PTSD (*n* = 40)	5%	4 items of the BSI (SI), PSQI PTSD addendum modified (nightmares) PCL-5 (PTSD)	- OSAS severity was directly related to SI - Depression was a significant mediator in the relationship between RDI and SI
*Krakow et al. (43)*	Prevalence of sleep disorders and the influence on suicidality, and depression severity	Female sexual assault survivors enrolled in a nightmare-treatment program (*n* = 153), with PTSD: 94%	0%	Wisconsin Cohort Sleep Survey (sleep disorders), Nightmare Frequency Questionnaire (nightmares), PSQI (sleep quality), PSS (PTSD)	- Prevalence of sleep breathing disorder: 15% - Prevalence of sleep movement disorder: 29,4% - Association of potential sleep disorders with greater depression and greater suicidality - Prevalence of combination of both disorders: 35,9%; this group suffered from most severe depression and suicidality

*Cursive references denotes studies on civilian samples*.

### Sleep Disturbances (Insomnia)

The respective studies are listed in Table 1. One study in military personnel investigated the relationship between insomnia symptoms and suicidal ideation and behavior after controlling for depressive symptom severity, hopelessness, PTSD diagnosis, anxiety symptoms, and drug and alcohol abuse in a cross-sectional as well as longitudinal design (54). It was shown that insomnia symptoms were cross-sectionally associated with suicidal ideation. Using a longitudinal design, insomnia symptoms were unique predictors of suicide attempts after controlling for baseline self-insomnia, depressive symptoms and hopelessness (54). The study is often cited as the prime example of a close link between sleep disturbances and suicidality independent of the presence of depression in PTSD. In addition, another study in veterans found that the association of sleep disturbance with suicidal ideations remained significant after controlling for age, alcohol dependence, depression, and PTSD (49). This was replicated in another study on veterans for poor sleep quality in general, but not for insomnia symptoms (51). A chart review provided evidence for a longitudinal relationship between sleep and suicidality; veterans with sleep disturbance died sooner after their last visit compared to those without sleep disturbance, even after adjusting for the presence of mental health or substance use symptoms (53).

New findings with respect to the longitudinal relationship revealed that pre-deployment insomnia was associated with increased risk of post-deployment PTSD and suicidal ideation even after adjusting for sociodemographic characteristics and prior deployment history (70). This is in line with prior findings. Sleep disturbance is not only a symptom of PTSD, but when existing prior to trauma they may also be a risk factor for developing PTSD in civilians (74) and in military personnel (75–77).

Consistent with these findings, an analysis of a large suicide attempt database from the U.S. Department of Veterans Affairs also revealed an independent association between insomnia and suicide attempts even after controlling for several socio-demographic factors and diverse psychiatric disorders. Furthermore, sleep medicine visits 180 days prior to the index date were associated with a decreased likelihood of suicide attempt for individuals with sleep disorders. Thus, the assessment and treatment of sleep disorders should be considered in context of strategies to augment suicide prevention efforts (56).

A proof-of-concept randomized clinical trial investigated the effects of a brief cognitive behavioral therapy for insomnia in primary care patients with suicidal ideation and insomnia in addition to either major depressive disorder and/or PTSD. The effect size of brief cognitive behavioral therapy for insomnia on suicidal ideation intensity was not significant. Effects were large on insomnia and depression with no effect on PTSD (66).

A birth cohort study in young Australians (the only study with non-veterans in this context) showed that PTSD symptoms indirectly predicted suicidal ideation via comorbid sleep disturbances even when adjusting for major depression symptoms, poly-victimization and gender. Notably, poly-victimization predicted sleep disturbances and suicidal ideation independently of PTSD or major depression (46).

### Nightmares

The studies focusing on nightmares are listed in Table 2. Two studies focused on the association between suicidality and nightmares in the context of PTSD. In the first study, the authors reported that trauma-related nightmares were highly prevalent (67.9%) but not associated with suicidal ideations. However, in a regression model, the presence of probable PTSD was significantly associated with suicidal ideations, indicating a mediating role of PTSD in the association between nightmares and suicidal ideations. Probable major depressive disorder emerged as the strongest predictor of suicidal ideation (52). These results were confirmed in a subsequent study by the same authors (59). Recently, corresponding results regarding the association between suicide attempts and nightmares were reported in a sample with veterans. After controlling for psychiatric disorders, nightmares were no longer significant associated with suicidal attempts (56).

In contrast to these findings, in a sample of trauma-exposed civilians with PTSD symptoms, nightmares were both directly and indirectly associated with suicidal behavior, through perceptions of defeat, entrapment, and hopelessness, independent of comorbid insomnia and depression (71). Further analyses supported that the relationship between nightmares and suicidal behavior was partially mediated by a multistep pathway via defeat, entrapment, and hopelessness (71).

Prazosin, an α1-adrenergic receptor antagonist, has been effective in alleviating nightmares associated with PTSD in military veterans (78, 79). However, in a randomized clinical trial of prazosin in suicidal PTSD patients there was no significant improvement of suicidal ideation or day time-only PTSD symptoms. In addition, nightmares and insomnia showed significantly less improvement in the prazosin group (72). These findings are in line with a recent multi-center randomized trial on the effect of prazosin in veterans with chronic PTSD, which revealed no effects of prazosin on nightmares or sleep quality (73). Of note, fewer patients in the prazosin group reported suicidal ideation compared with the placebo group.

### Sleep-Related Breathing Disorders

The studies focused on sleep-related breathing disorders are listed in Table 3. A sample of female sexual assault survivors with PTSD was assessed for subjectively determined sleep related breathing and movement disorders and 80% of the participants presented with such disorders (43). Participants with potential sleep disorders suffered from a higher degree of depression and suicidality in comparison to those without any potential sleep disorder. It was hypothesized that this effect could be mediated through chronic sleep fragmentation. The study was limited insofar as the participants were recruited through a nightmare-treatment program. Nightmares are estimated as a marker for other sleep disorders, therefore nightmares could increase the total prevalence of sleep disorders (43). In a study performed to elucidate the association between suicidal ideations and OSAS, it was found that OSAS severity was directly related to suicidal ideation in (predominately female) PTSD patients who underwent a home sleep apnea monitoring. Depression was a significant mediator in the relationship between respiratory disturbance index and suicidal ideation (80).

### Sleep Duration

Sleep duration itself is purported to have an effect on suicidality (5, 6, 81). In redeployed soldiers, after controlling for combat exposure, short sleep duration was associated with symptoms of depression, PTSD, and panic syndrome, and with high-risk health behaviors such as abuse of tobacco and alcohol products, as well as suicide attempts. Short sleep duration emerged as a significant predictor of suicide risk; conversely, short sleep was the strongest predictor of PTSD symptoms (63). Another study in veterans corroborated an association between sleep duration, very short sleep duration (≤5 h of sleep) as well as long sleep duration (≥9 h): after adjusting for covariates, both conditions were associated with increased odds of current PTSD and major depression, but not with suicidal ideation. However, poor sleep quality was associated with PTSD and suicidal ideation as well as with panic disorder, major depressive disorder, and risky drinking (55).

### Circadian Aspects

We identified one study in civilian patients with PTSD investigating the effect of alterations in rhythmicity and vegetative function including sleep disturbances as correlates of suicidality. As a result, all lifetime mood spectrum sub-domains (rhythmicity, sleep, appetite/weight, sexual function, physical symptoms) were associated with an increased likelihood of suicidal ideation. Interestingly, another study reported that an eveningness chronotype in PTSD was associated with an increased likelihood of suicidal ideations, but not suicide attempts (47).

## Mediating Factors

Various mediating factors may have an influence on the association between sleep disturbances, suicidality, and PTSD symptoms.

### Depression

A study on deployed soldiers with mild trauma brain injury showed a significant association between increased suicidality and depression as well as the interaction of depression with PTSD symptoms. Interestingly, longer duration of loss of consciousness was associated with decreased likelihood for any suicidality (50). Another study found an association between sleep disturbances with concurrent and prospective suicide ideation in three active military samples. When adjusting for age, gender, depression, and PTSD, insomnia severity was no longer directly associated with suicidal ideation either concurrently or prospectively, whereby depression mediated the relation of insomnia severity with suicide risk (58). One study examined the association of suicidal ideation and sleep disturbances after controlling for probable PTSD, depression, alcohol use disorder (AUD) and generalized anxiety disorder. Neither sleep disturbances nor nightmares significantly predicted suicidal ideation; instead, major depression emerged as the most significant predictor (52). Depression also mediated the relationship between insomnia and nightmares and suicidal ideations (59). Another study investigated the associations among insomnia symptoms, PTSD, and depressive symptoms, interpersonal theory of suicide variables, and risk for suicidal behavior in community veterans and confirmed a mediating effect of depression. In extension to this, PTSD emerged also as a mediator between insomnia and suicidal ideation. The interpersonal theory of suicide variables of thwarted belongingness and perceived burdensomeness mediated the association of depressive and PTSD symptoms with risk for suicidal behavior, indicating a sequential association (57).

### Other Mediators

In military service members with PTSD, perceived stress mediated the relationship between sleep issues and suicidal ideation; however, after accounting for mental health symptoms (depression, perceived stress, PTSD symptoms) sleep no longer had a significant direct effect on suicidal ideation (65). In outpatient veterans misusing alcohol the relationship between insomnia symptoms and suicidal ideation was no longer significant after controlling for other mental disorders. However, poor sleep quality remained significantly associated with increased suicidal ideation even after controlling for other risk factors (51). Among suicidal military inpatients, those with PTSD reported more disturbed sleep and reduced social and work adjustment. Still, this association between functionality and PTSD status was no longer significant after adjusting for gender and psychiatric comorbidity as individuals. Patients with a higher number of psychiatric comorbidities demonstrated a higher likelihood of meeting PTSD criteria (67).

Beyond mental health conditions in general, some studies also investigated specific mediators, i.e., AUD (62), agitation (60), and exercise (48) in the context of PTSD, suicidality, and sleep disturbances. Given that AUD und PTSD frequently co-occur (82), evidence for a mediating role of AUD and insomnia in the relationship between PTSD symptoms and suicidal ideation derived from a large sample of Korean firefighters (62). With respect to a moderating role of agitation within the relationship between insomnia and current suicidal ideations, a study on U.S. military personnel showed significant effects only at high levels of agitation. PTSD symptoms, depressive symptoms, and lifetime number of suicide attempts were each associated with greater levels of agitation, insomnia, and current suicidal ideation (60).

## PTSD Symptom Clusters and Suicidality

A burgeoning approach to determine suicide risk in the context of PTSD is the analysis of specific PTSD symptom clusters (69, 83–90) yielding conflicting results. Considering sleep disturbances and nightmares as diagnostic criteria for PTSD, a within-cluster item analysis would be necessary. So far, only one study meets this requirement: reporting that an increased number of re-experiencing and avoidance symptoms significantly correlated with suicide attempts. In addition, a significant association between nightmares and suicide attempts was found; however, this effect was no longer present after controlling for covariates (69). In this study, sleep disturbances (85.0%) and nightmares (77.8%) were highly prevalent, but were evaluated by using a single, dichotomous question only, indicating a possible lack of selectivity. To date, only three studies examined the relationship between PTSD symptom clusters and suicidality in a prospective design (88, 90, 91), with two of them pointing at a unique relationship between alterations in arousal and reactivity and higher suicidality (90, 91). Unfortunately, sleep disturbance as a feature of the cluster “alterations in arousal and reactivity” was not separately considered in these studies. Thus, it is not possible to state, whether sleep disturbances had a significant influence on these results.

A more tailored approach on individual PTSD symptoms a priori has been provided by the use of a more detailed method for the assessment, monitoring, and treatment of PTSD symptoms (61). This study of predominantly trauma-exposed military veterans investigated the association between individual symptoms of PTSD and measures of functioning, quality of life, and suicidal ideation. Among others, sleep difficulties explained problems in physical functioning and quality of life. These findings persisted after adjustment for lifetime trauma burden and severity of PTSD and depressive symptoms. No association was found between suicidal ideations and sleep difficulties (61). However, it should be emphasized that only about 10% of the sample met full PTSD criteria.

## Discussion

The present studies revealed heterogeneous and partially contradictory results. A direct association between sleep disturbances and suicidal behavior in patients with PTSD has been reported by a few studies (49, 51, 53, 54, 70),while others found no association after controlling for covariates such as depression (50, 52, 57–59). In a sample of veterans with PTSD and two or more comorbid disorders there was a higher risk of suicidal ideation compared to veterans with PTSD only (92).

The study results should be interpreted with caution because differences in approach and samples, as well as different applied measurements and outcome parameters, hinder direct comparisons. In particular, due to the studies available, various types of sleep disturbances, measured by using different methodologies, were included. Some studies focused on specific sleep disorders such as insomnia, nightmares or sleep related breathing disorders (57, 80, 93) while others measured only sleep quality or sleep duration in a more general approach (43, 48) or used a single sleep item only (59, 69) limiting the comparability. In addition, almost all studies used self-report instruments to assess sleep variables. However, subjective and objective measurements of sleep may differ also in PTSD patients and do not necessarily co-vary (94, 95). The use of sleep diaries could be a more reliable measurement in order to assess prospectively sleep disorders compared to a selective survey (96). The vast majority of studies used a cross-sectional design, a major limitation for drawing causal conclusions. With respect to suicidality, it can be stated that the outcome parameters vary between suicidal ideation (60), suicidal behavior (57), suicide attempts (56), death by suicide (53), and through combinations of those (51). Thus, “suicidality” does not always carry the same meaning and precludes comparability.

Of importance, an explicit PTSD diagnosis was a primary inclusion criterion only in five studies (27, 47, 48, 72, 73), whereas in all other studies a “possible” PTSD diagnosis was recorded and considered in data analysis with prevalence rates between 3.6% (62) and 94% (43). Therefore, the absolute number of subjects with PTSD was considerably lower than the absolute number of study participants, mitigating the statistical validity. In military samples, the vast majority of participants were men with a proportion of at least 69.6% (57) up to 99.7% (53). In civilian samples, women were overrepresented by trend with a proportion of at least 49.2% (47) up to 100% (43). This remarkable disproportion in gender ratios should be taken into account in light of gender differences in suicidal behavior among persons with PTSD. For example, among veterans with PTSD, women have a lower risk of dying by suicide compared with men (97).

In addition, most studies have included military personnel and veterans. A large cohort study, for example, revealed an association between type and number of traumata with suicidal ideation and suicide attempts in patients with PTSD (98). Among others, peacekeeping traumata had the highest rates of suicidal ideation and suicidal behavior. In the civilian population, however, a broader spectrum of trauma types may occur. Moreover, it remains questionable whether results obtained from veterans can be transferred to the civilian population. Evaluating the risk of death by suicide in military personnel, some specific aspects have to be noted; historically, soldiers have had a markedly lower suicide rate than civilians (99, 100). However, since 2005 the incidence of suicide in Army and Marine personnel has nearly doubled and remained elevated (101, 102). Several studies showed an increased risk of people with PTSD dying by suicide (103–106), while others revealed a decreased risk of death by suicide in military personnel (107–110). The background of these confusing results is discussed in detail in a recently published review (111).

The current overview has some limitations. Most importantly, it does not meet the requirements of a systematic review because we did not perform a systematic literature research. In addition, we had no strict inclusion/exclusion criteria to identify relevant studies. In many studies, the relationship between sleep disturbances, suicidality, and PTSD was a minor issue and evaded a literature search using Medical Subject Headings terms. Therefore, the overview may not cover all studies in the field. Nevertheless, diagnosing and treating sleep disturbances early in the context of trauma exposition may provide preventive strategies regarding the development of PTSD. For example, non-drug interventions such as cognitive behavioral therapy for insomnia has been shown to reduce sleep disturbances and, subsequently, PTSD symptoms (112). It remains to be clarified whether or not suicidal behavior in PTSD can also be improved by treating impaired sleep.

## Conclusion

Heterogeneous study approaches, different samples and applied measurements, and diverse outcome parameters hinder a direct comparison of studies examining sleep disturbances, suicidality, and PTSD. In addition, due to limited study methodologies, a causal relationship in these entities cannot be shown. Future research including adequately designed studies is necessary to clarify the complex relationship between these parameters. Particularly, more studies in the civilian population are needed also to tackle the value of treatment of sleep disturbances for suicide prevention in PTSD.

## Author Contributions

FW and TW have made substantial contributions to conception and design of the study and analyzed the data. FW executed the acquisition of data. FW, CN, and TW have been involved in the interpretation of data, drafting and revising the manuscript for important intellectual content. All authors have read and approved the final manuscript.

### Conflict of Interest

The authors declare that the research was conducted in the absence of any commercial or financial relationships that could be construed as a potential conflict of interest. The reviewer RG declared a past co-authorship with the authors to the handling editor.

## Abbreviations

AIS: Athens Insomnia Scale
AOR: Adjusted odds ratio
AUD: Alcohol use disorder
bCBTi: Brief cognitive behavioral therapy for insomnia
BDI II: Beck Depression Inventory, 2. Edition
BSSI: Beck Scale for Suicidal Ideation
CAF: Canadian Armed Force
CAPS: Clinician-Administered PTSD Scale for DSM-IV
CIDI-Auto: Composite International Diagnostic Interview, version 2.4
C-SSRS: Columbia Suicide Severity Rating Scale
DDNSI: Disturbing Dreams and Nightmare Severity Index
DSI-SS: Depressive Symptom Index-Suicidality Subscale
DSM-IV: Diagnostic and Statistical Manual of Mental Disorders, 4. Version
GAD: Generalized anxiety disorder
HRA II: Health Risk Appraisal
IPT: Interpersonal Theory of Suicide
ISI: Insomnia Severity Index
LSD: Long sleep duration
MDD: Major depressive disorder
MINI: Mini International Neuropsychiatric Screen and Interview
MOODS-SR: Mood Spectrum-Self Report
MSSI: Modified Scale for Suicidal Ideationl
mTBI: Mild traumatic brain injury
MUSP: Mater University Study of Pregnancy
NESARC: National Epidemiologic Survey on Alcohol and Related Conditions
OIF: Operation Iraqi Freedom
OR: Odds ratio
OSAS: Obstructive sleep apnea syndrome
PCL: Posttraumatic Stress Disorder Symptoms Checklist
PCL-5: Posttraumatic Stress Disorder Checklist for DSM-5
PCL-C: Posttraumatic Stress Disorder Symptoms Checklist—Civilian version
PCL-M: Posttraumatic Stress Disorder Symptoms Checklist—Military version
PC-PTSD: Primary Care PTSD Screen
PD: Panic disorder
PHQ-9: Patient Health Questionnaire 9
PROMIS: Patient-Reported Outcomes Measurement Information System
PSI: Paykel Suicide Items
PSQI: Pittsburgh Sleep Quality Index
PSQI-A: Pittsburgh Sleep Quality Index-Addendum
PSS: PTSD Symptom Scale
PTSD: Posttraumatic stress disorder
RCT: Randomized clinical trial
RDI: Respiratory disturbance index
REM: Rapid eye movement
SA: Suicide attempt
SB: Suicidal behavior
SBQ-R: Suicidal Behaviors Questionnaire-Revised
SCIDI/P: Structured Clinical Interview for DSMIV-TR Axis I Disorders
SI: Suicidal ideation
SSD: Short sleep duration
SSI: Scale for Suicide Ideation
SWS: Suicide warning signs survey
TAU: Treatment as usual
VHA: Veterans Health Administration.

## References

[1] WHO Mental Health Prevention of Suicidal Behaviours: A Task for All. (2015). Available online at: http://www.who.int/mental_health/prevention/suicide/background (accessed January 30, 2020).

[2] McCall WV Black CG. The link between suicide and insomnia: theoretical mechanisms. Curr Psychiatry Rep. (2013) 15:389. 10.1007/s11920-013-0389-9 23949486 PMC3791319

[3] Bernert RA Kim JS Iwata NG Perlis ML. Sleep disturbances as an evidence-based suicide risk factor. Curr Psychiatry Rep. (2015) 17:15. 10.1007/s11920-015-0554-4 25698339 PMC6613558

[4] Liu JW Tu YK Lai YF Lee HC Tsai PS Chen TJ. Associations between sleep disturbances and suicidal ideation, plans, and attempts in adolescents: a systematic review and meta-analysis. Sleep Med Rev. (2019) 42:119–26. 10.1093/sleep/zsz054 30843072

[5] Lee J Jang H Kim J Min S. Development of a suicide index model in general adolescents using the South Korea 2012-2016 national representative survey data. Sci Rep. (2019) 9:1846. 10.1038/s41598-019-38886-z 30755681 PMC6372692

[6] Littlewood DL Kyle SD Carter LA Peters S Pratt D Gooding P. Short sleep duration and poor sleep quality predict next-day suicidal ideation: an ecological momentary assessment study. Psychol Med. (2019) 49:403–11. 10.1017/S0033291718001009 29697037 PMC6331731

[7] Rössler W Angst J Ajdacic-Gross V Haker H Berrouiguet S Ujeyl M. Sleep disturbances and suicidality-a longitudinal analysis from a representative community study over 30 years. Front Psychiatry. (2018) 9:320. 10.3389/fpsyt.2018.00320 30061849 PMC6054984

[8] Russell K Rasmussen S Hunter SC. Insomnia and nightmares as markers of risk for suicidal ideation in young people: investigating the role of defeat and entrapment. J Clin Sleep Med. (2018) 14:775–84. 10.5664/jcsm.7104 29734987 PMC5940428

[9] Bernert RA Joiner TE Cukrowicz KC Schmidt NB Krakow B. Suicidality and sleep disturbances. Sleep. (2005) 28:1135–41. 10.1093/sleep/28.9.1135 16268383

[10] Tae H Jeong BR Chae JH. Sleep problems as a risk factor for suicide: Are certain specific sleep domains associated with increased suicide risk? J Affect Disord. (2019) 252:182–9. 10.1016/j.jad.2019.04.053 30986733

[11] Sjöström N Hetta J Waern M. Persistent nightmares are associated with repeat suicide attempt: a prospective study. Psychiatry Res. (2009) 170:208–11. 10.1016/j.psychres.2008.09.006 19900715

[12] Tanskanen A Tuomilehto J Viinamäki H Vartiainen E Lehtonen J Puska P. Nightmares as predictors of suicide. Sleep. (2001) 24:844–7. 10.1093/sleep/24.7.845 11683487

[13] Sandman N Valli K Kronholm E Vartiainen E Laatikainen T Paunio T. Nightmares as predictors of suicide: an extension study including war veterans. Sci Rep. (2017) 7:44756. 10.1038/srep44756 28294195 PMC5353666

[14] Titus CE Speed KJ Cartwright PM Drapeau CW Heo Y Nadorff MR. What role do nightmares play in suicide? A brief exploration. Curr Opin Psychol. (2018) 22:59–62. 10.1016/j.copsyc.2017.08.022 28846873

[15] Nadorff MR Nadorff DK Germain A. Nightmares: under-reported, undetected, and therefore untreated. J Clin Sleep Med. (2015) 11:747–50. 10.5664/jcsm.4850 25845898 PMC4481058

[16] Kessler RC Petukhova M Sampson NA Zaslavsky AM Wittchen H-U. Twelve-month and lifetime prevalence and lifetime morbid risk of anxiety and mood disorders in the United States. Int J Methods Psychiatr Res. (2012) 21:169–84. 10.1002/mpr.1359 22865617 PMC4005415

[17] Kessler RC Ruscio AM Shear K Wittchen HU. Epidemiology of anxiety disorders. Curr Top Behav Neurosci. (2010) 2:21–35. 10.1007/7854_2009_9 21309104

[18] Ross RJ Ball WA Sullivan KA Caroff SN. Sleep disturbance as the hallmark of posttraumatic stress disorder. Am J Psychiatry. (1989) 146:697–707. 10.1176/ajp.146.1.128-a2658624

[19] Wittmann L Schredl M Kramer M. Dreaming in posttraumatic stress disorder: a critical review of phenomenology, psychophysiology and treatment. Psychother Psychosom. (2007) 76:25–39. 10.1159/000096362 17170561

[20] Ohayon MM Shapiro CM. Sleep disturbances and psychiatric disorders associated with posttraumatic stress disorder in the general population. Compr Psychiatry. (2000) 41:469–78. 10.1053/comp.2000.16568 11086154

[21] Spoormaker VI Montgomery P. Disturbed sleep in post-traumatic stress disorder: secondary symptom or core feature? Sleep Med Rev. (2008) 12:169–84. 10.1016/j.smrv.2007.08.008 18424196

[22] Miller KE Brownlow JA Gehrman PR. Sleep in PTSD: treatment approaches and outcomes. Curr Opin Psychol. (2019) 34:12–7. 10.1016/j.copsyc.2019.08.017 31541965 PMC7337559

[23] Germain A Buysse DJ Nofzinger E. Sleep-specific mechanisms underlying posttraumatic stress disorder: integrative review and neurobiological hypotheses. Sleep Med Rev. (2008) 12:185–95. 10.1016/j.smrv.2007.09.003 17997114 PMC2490669

[24] Miller KE Brownlow JA Woodward S Gehrman PR. Sleep and dreaming in posttraumatic stress disorder. Curr Psychiatry Rep. (2017) 19:74. 10.1007/s11920-017-0827-1 28828641

[25] El-Solh AA Riaz U Roberts J. Sleep disorders in patients with posttraumatic stress disorder. Chest. (2018) 154:427–39. 10.1016/j.chest.2018.04.007 29684315

[26] Krakow BJ Ulibarri VA Moore BA McIver ND. Posttraumatic stress disorder and sleep-disordered breathing: a review of comorbidity research. Sleep Med Rev. (2015) 24:37–45. 10.1016/j.smrv.2014.11.001 25644985

[27] Gupta MA Simpson FC. Obstructive sleep apnea and psychiatric disorders: a systematic review. J Clin Sleep Med. (2015) 11:165–75. 10.5664/jcsm.4466 25406268 PMC4298774

[28] Orr JE Smales C Alexander TH Stepnowsky C Pillar G Malhotra A. Treatment of OSA with CPAP is associated with improvement in PTSD symptoms among veterans. J Clin Sleep Med. (2017) 13:57–63. 10.5664/jcsm.6388 27707436 PMC5181615

[29] El-Solh AA Adamo D Kufel T. Comorbid insomnia and sleep apnea in Veterans with post-traumatic stress disorder. Sleep Breath. (2018) 22:23–31. 10.1007/s11325-017-1618-y 29330769

[30] Mysliwiec V O'Reilly B Polchinski J Kwon HP Germain A Roth BJ. Trauma associated sleep disorder: a proposed parasomnia encompassing disruptive nocturnal behaviors, nightmares, and REM without atonia in trauma survivors. J Clin Sleep Med. (2014) 10:1143–8. 10.5664/jcsm.4120 25317096 PMC4173093

[31] Brock MS Powell TA Creamer JL Moore BA Mysliwiec V. Trauma associated sleep disorder: clinical developments 5 years after discovery. Curr Psychiatry Rep. (2019) 21:80. 10.1007/s11920-019-1066-4 31410580

[32] Bernal M Haro JM Bernert S Brugha T Graaf R de Bruffaerts R. Risk factors for suicidality in Europe: results from the ESEMED study. J Affect Disord. (2007) 101:27–34. 10.1016/j.jad.2006.09.018 17074395

[33] Nock MK Hwang I Sampson N Kessler RC Angermeyer M Beautrais A. Cross-national analysis of the associations among mental disorders and suicidal behavior: findings from the WHO World Mental Health Surveys. PLoS Med. (2009) 6:e1000123. 10.1371/journal.pmed.1000123 19668361 PMC2717212

[34] Panagioti M Gooding PA Tarrier N. A meta-analysis of the association between posttraumatic stress disorder and suicidality: the role of comorbid depression. Compr Psychiatry. (2012) 53:915–30. 10.1016/j.comppsych.2012.02.009 22483367

[35] Panagioti M Gooding P Tarrier N. Post-traumatic stress disorder and suicidal behavior: a narrative review. Clin Psychol Rev. (2009) 29:471–82. 10.1016/j.cpr.2009.05.001 19539412

[36] Gradus JL. Prevalence and prognosis of stress disorders: a review of the epidemiologic literature. Clin Epidemiol. (2017) 9:251–60. 10.2147/CLEP.S106250 28496365 PMC5422316

[37] Krysinska K Lester D. Post-traumatic stress disorder and suicide risk: a systematic review. Arch Suicide Res. (2010) 14:1–23. 10.1080/13811110903478997 20112140

[38] Kessler RC Borges G Walters EE. Prevalence of and risk factors for lifetime suicide attempts in the National Comorbidity Survey. Arch Gen Psychiatry. (1999) 56:617–26. 10.1001/archpsyc.56.7.617 10401507

[39] Gradus JL Qin P Lincoln AK Miller M Lawler E Sørensen HT. Posttraumatic stress disorder and completed suicide. Am J Epidemiol. (2010) 171:721–7. 10.1093/aje/kwp456 20160171

[40] Bryan CJ Grove JL Kimbrel NA. Theory-driven models of self-directed violence among individuals with PTSD. Curr Opin Psychol. (2017) 14:12–7. 10.1016/j.copsyc.2016.09.007 28813309

[41] May AM Klonsky ED What distinguishes suicide attempters from suicide ideators? A meta-analysis of potential factors. Clin Psychol Sci Pract. (2016) 23:5–20. 10.1111/cpsp.12136

[42] Pigeon WR Pinquart M Conner K. Meta-analysis of sleep disturbance and suicidal thoughts and behaviors. J Clin Psychiatry. (2012) 73:e1160–7. 10.4088/JCP.11r07586 23059158

[43] Krakow B Artar A Warner TD Melendrez D Johnston L Hollifield M. Sleep disorder, depression, and suicidality in female sexual assault survivors. Crisis. (2000) 21:163–70. 10.1027//0227-5910.21.4.163 11419527

[44] Malik S Kanwar A Sim LA Prokop LJ Wang Z Benkhadra K. The association between sleep disturbances and suicidal behaviors in patients with psychiatric diagnoses: a systematic review and meta-analysis. Syst Rev. (2014) 3:18. 10.1186/2046-4053-3-18 24568642 PMC3945796

[45] Pigeon WR Bishop TM Titus CE The relationship between sleep disturbance, suicidal ideation, suicide attempts, and suicide among adults: a systematic review. Psychiatr Ann. (2016) 46:177–86. 10.3928/00485713-20160128-01

[46] Betts KS Williams GM Najman JM Alati R. The role of sleep disturbance in the relationship between post-traumatic stress disorder and suicidal ideation. J Anxiety Disord. (2013) 27:735–41. 10.1016/j.janxdis.2013.09.011 24176805

[47] Dell'Osso L Massimetti G Conversano C Bertelloni CA Carta MG Ricca V. Alterations in circadian/seasonal rhythms and vegetative functions are related to suicidality in DSM-5 PTSD. BMC Psychiatry. (2014) 14:352. 10.1186/s12888-014-0352-2 25496184 PMC4297401

[48] Davidson CL Babson KA Bonn-Miller MO Souter T Vannoy S. The impact of exercise on suicide risk: examining pathways through depression, PTSD, and sleep in an inpatient sample of veterans. Suicide Life Threat Behav. (2013) 43:279–89. 10.1111/sltb.12014 23901428

[49] Bishop TM Pigeon WR Possemato K Sleep disturbance and its association with suicidal ideation in veterans. Mil Behav Health. (2013) 1:81–4. 10.1080/21635781.2013.830061

[50] Bryan CJ Clemans TA Hernandez AM Rudd MD. Loss of consciousness, depression, posttraumatic stress disorder, and suicide risk among deployed military personnel with mild traumatic brain injury. J Head Trauma Rehabil. (2013) 28:13–20. 10.1097/HTR.0b013e31826c73cc 23076097

[51] Chakravorty S Grandner MA Mavandadi S Perlis ML Sturgis EB Oslin DW. Suicidal ideation in veterans misusing alcohol: relationships with insomnia symptoms and sleep duration. Addict Behav. (2014) 39:399–405. 10.1016/j.addbeh.2013.09.022 24169371 PMC4406056

[52] Don Richardson J St. Cyr K Nelson C Elhai JD Sareen J. Sleep disturbances and suicidal ideation in a sample of treatment-seeking Canadian Forces members and veterans. Psychiatry Res. (2014) 218:118–23. 10.1016/j.psychres.2014.04.008 24755040

[53] Pigeon WR Britton PC Ilgen MA Chapman B Conner KR. Sleep disturbance preceding suicide among veterans. Am J Public Health. (2012) 102 (Suppl. 1):S93–7. 10.2105/AJPH.2011.300470 22390611 PMC3496468

[54] Ribeiro JD Pease JL Gutierrez PM Silva C Bernert RA Rudd MD. Sleep problems outperform depression and hopelessness as cross-sectional and longitudinal predictors of suicidal ideation and behavior in young adults in the military. J Affect Disord. (2012) 136:743–50. 10.1016/j.jad.2011.09.049 22032872

[55] Swinkels CM Ulmer CS Beckham JC Buse N Calhoun PS. The association of sleep duration, mental health, and health risk behaviors among US Afghanistan/Iraq Era veterans. Sleep. (2013) 36:1019–25. 10.5665/sleep.2800 23814338 PMC3669061

[56] Bishop TM Walsh PG Ashrafioun L Lavigne JE Pigeon WR. Sleep, suicide behaviors, and the protective role of sleep medicine. Sleep Med. (2019) 66:264–70. 10.1016/j.sleep.2019.07.016 31727433

[57] Britton PC McKinney JM Bishop TM Pigeon WR Hirsch JK. Insomnia and risk for suicidal behavior: a test of a mechanistic transdiagnostic model in veterans. J Affect Disord. (2019) 245:412–8. 10.1016/j.jad.2018.11.044 30423469

[58] Bryan CJ Gonzales J Rudd MD Bryan AO Clemans TA Ray-Sannerud B. Depression mediates the relation of insomnia severity with suicide risk in three clinical samples of U. S military personnel Depress Anxiety. (2015) 32:647–55. 10.1002/da.22383 26047362

[59] Don Richardson J King L St Cyr K Shnaider P Roth ML Ketcheson F. Depression and the relationship between sleep disturbances, nightmares, and suicidal ideation in treatment-seeking Canadian Armed Forces members and veterans. BMC Psychiatry. (2018) 18:204. 10.1186/s12888-018-1782-z 29921268 PMC6011186

[60] Fisher K Houtsma C Assavedo BL Green BA Anestis MD. Agitation as a moderator of the relationship between insomnia and current suicidal ideation in the military. Arch Suicide Res. (2017) 21:531–43. 10.1080/13811118.2016.1193077 27435680

[61] Kachadourian LK Harpaz-Rotem I Tsai J Southwick SM Pietrzak RH Posttraumatic stress disorder symptoms, functioning, and suicidal ideation in, U. S, Military Veterans: a symptomics approach. Prim Care Companion CNS Disord. (2019) 21:18m02402 10.4088/PCC.18m02402 31050228

[62] Kim JI Park H Kim J-H. Alcohol use disorders and insomnia mediate the association between PTSD symptoms and suicidal ideation in Korean firefighters. Depress Anxiety. (2018) 35:1095–103. 10.1002/da.22803 30028563

[63] Luxton DD Greenburg D Ryan J Niven A Wheeler G Mysliwiec V. Prevalence and impact of short sleep duration in redeployed OIF soldiers. Sleep. (2011) 34:1189–95. 10.5665/SLEEP.1236 21886356 PMC3157660

[64] McClure JR Criqui MH Macera CA Ji M Nievergelt CM Zisook S. Prevalence of suicidal ideation and other suicide warning signs in veterans attending an urgent care psychiatric clinic. Compr Psychiatry. (2015) 60:149–55. 10.1016/j.comppsych.2014.09.010 25799463

[65] Morgan JK Hourani L Tueller S Strange L Lane ME Lewis GF Effects of sleep issues on suicidal ideation in a military sample: the mediating role of mental health. Mil Behav Health. (2018) 6:234–42. 10.1080/21635781.2017.1406415

[66] Pigeon WR Funderburk JS Cross W Bishop TM Crean HF. Brief CBT for insomnia delivered in primary care to patients endorsing suicidal ideation: a proof-of-concept randomized clinical trial. Transl Behav Med. (2019) 9:1169–77. 10.1093/tbm/ibz108 31271210

[67] Ribeiro SP LaCroix JM Oliveira F de Novak LA Lee-Tauler SY Darmour CA. The link between posttraumatic stress disorder and functionality among united states military service members psychiatrically hospitalized following a suicide crisis. Healthcare. (2018) 6:1–13. 10.3390/healthcare6030095 30087239 PMC6164520

[68] Richardson JD Thompson A King L Corbett B Shnaider P St Cyr K. Insomnia, psychiatric disorders and suicidal ideation in a national representative sample of active Canadian forces members. BMC Psychiatry. (2017) 17:211. 10.1186/s12888-017-1372-5 28583100 PMC5460415

[69] Selaman ZMH Chartrand HK Bolton JM Sareen J. Which symptoms of post-traumatic stress disorder are associated with suicide attempts? J Anxiety Disord. (2014) 28:246–51. 10.1016/j.janxdis.2013.12.005 24507633

[70] Wang HE Campbell-Sills L Kessler RC Sun X Heeringa SG Nock MK. Pre-deployment insomnia is associated with post-deployment post-traumatic stress disorder and suicidal ideation in, US, Army soldiers. Sleep. (2019) 42:1–9. 10.1093/sleep/zsy229 30508139 PMC6369721

[71] Littlewood DL Gooding PA Panagioti M Kyle SD. Nightmares and suicide in posttraumatic stress disorder: the mediating role of defeat, entrapment, and hopelessness. J Clin Sleep Med. (2016) 12:393–9. 10.5664/jcsm.5592 26564386 PMC4773636

[72] McCall WV Pillai A Case D McCloud L Nolla T Branch F. A pilot, randomized clinical trial of bedtime doses of prazosin versus placebo in suicidal posttraumatic stress disorder patients with nightmares. J Clin Psychopharmacol. (2018) 38:618–21. 10.1097/JCP.0000000000000968 30335633

[73] Raskind MA Peskind ER Chow B Harris C Davis-Karim A Holmes HA. Trial of prazosin for post-traumatic stress disorder in military veterans. N Engl J Med. (2018) 378:507–17. 10.1056/NEJMoa1507598 29414272

[74] Bryant RA Creamer M O'Donnell M Silove D McFarlane AC. Sleep disturbance immediately prior to trauma predicts subsequent psychiatric disorder. Sleep. (2010) 33:69–74. 10.1093/sleep/33.1.69 20120622 PMC2802249

[75] Milanak ME Zuromski KL Cero I Wilkerson AK Resnick HS Kilpatrick DG Traumatic event exposure, posttraumatic stress disorder, and sleep disturbances in a national sample of US adults. J Traumatic Stress. (2019) 32:14–22. 10.1002/jts.22360 PMC6814299 30702778

[76] Gehrman P Seelig AD Jacobson IG Boyko EJ Hooper TI Gackstetter GD. Predeployment sleep duration and insomnia symptoms as risk factors for new-onset mental health disorders following military deployment. Sleep. (2013) 36:1009–18. 10.5665/sleep.2798 23814337 PMC3669076

[77] van Liempt S van Zuiden M Westenberg H Super A Vermetten E. Impact of impaired sleep on the development of PTSD symptoms in combat veterans: a prospective longitudinal cohort study. Depress Anxiety. (2013) 30:469–74. 10.1002/da.22054 23389990

[78] Raskind MA Peterson K Williams T Hoff DJ Hart K Holmes H. A trial of prazosin for combat trauma PTSD with nightmares in active-duty soldiers returned from Iraq and Afghanistan. Am J Psychiatry. (2013) 170:1003–10. 10.1176/appi.ajp.2013.12081133 23846759

[79] Khachatryan D Groll D Booij L Sepehry AA Schütz CG. Prazosin for treating sleep disturbances in adults with posttraumatic stress disorder: a systematic review and meta-analysis of randomized controlled trials. Gen Hosp Psychiatry. (2016) 39:46–52. 10.1016/j.genhosppsych.2015.10.007 26644317

[80] Gupta MA Jarosz P. Obstructive sleep apnea severity is directly related to suicidal ideation in posttraumatic stress disorder. J Clin Sleep Med. (2018) 14:427–35. 10.5664/jcsm.6992 29510795 PMC5837844

[81] Goodwin RD Marusic A. Association between short sleep and suicidal ideation and suicide attempt among adults in the general population. Sleep. (2008) 31:1097–101. 10.5665/sleep/31.8.1097 18714781 PMC2542955

[82] Petrakis IL Simpson TL. Posttraumatic stress disorder and alcohol use disorder: a critical review of pharmacologic treatments. Alcohol Clin Exp Res. (2017) 41:226–37. 10.1111/acer.13297 28102573 PMC5375032

[83] Davis MT Witte TK Weathers FW Blevins CA The role of posttraumatic stress disorder symptom clusters in the prediction of passive suicidal ideation. Psychol Trauma. (2014) 6:S82–91. 10.1037/a0035966

[84] Bell JB Nye EC. Specific symptoms predict suicidal ideation in Vietnam combat veterans with chronic post-traumatic stress disorder. Mil Med. (2007) 172:1144–7. 10.7205/MILMED.172.11.1144 18062386

[85] Boffa JW Stanley IH Hom MA Norr AM Joiner TE Schmidt NB. PTSD symptoms and suicidal thoughts and behaviors among firefighters. J Psychiatr Res. (2017) 84:277–83. 10.1016/j.jpsychires.2016.10.014 27810667

[86] Zuromski KL Davis MT Witte TK Weathers F Blevins C. PTSD symptom clusters are differentially associated with components of the acquired capability for suicide. Suicide Life Threat Behav. (2014) 44:682–97. 10.1111/sltb.12098 24796870

[87] Guerra VS Calhoun PS. Examining the relation between posttraumatic stress disorder and suicidal ideation in an OEF/OIF veteran sample. J Anxiety Disord. (2011) 25:12–8. 10.1016/j.janxdis.2010.06.025 20709493 PMC3635678

[88] Horwitz AG Miron L Maieritsch KP. Prospective associations between DSM-5 PTSD symptom clusters and suicidal ideation in treatment-seeking veterans. Psychol Serv. (2019) 16:321–8. 10.1037/ser0000215 30359075

[89] Brown LA Contractor A Benhamou K. Posttraumatic stress disorder clusters and suicidal ideation. Psychiatry Res. (2018) 270:238–45. 10.1016/j.psychres.2018.09.030 30269041

[90] Panagioti M Angelakis I Tarrier N Gooding P. A prospective investigation of the impact of distinct posttraumatic (PTSD) symptom clusters on suicidal ideation. Cognit Ther Res. (2017) 41:645–53. 10.1007/s10608-016-9829-2 28751798 PMC5504127

[91] Stanley IH Rogers ML Hanson JE Gutierrez PM Joiner TE. PTSD symptom clusters and suicide attempts among high-risk military service members: a three-month prospective investigation. J Consult Clin Psychol. (2019) 87:67–78. 10.1037/ccp0000350 30431299

[92] Jakupcak M Cook J Imel Z Fontana A Rosenheck R McFall M. Posttraumatic stress disorder as a risk factor for suicidal ideation in Iraq and Afghanistan War veterans. J Trauma Stress. (2009) 22:303–6. 10.1002/jts.20423 19626682

[93] Littlewood DL Gooding PA Panagioti M Kyle SD. Investigating psychological mechanisms in relation to sleep problems and suicide. J Clin Sleep Med. (2016) 12:931. 10.5664/jcsm.5910 27250811 PMC4877330

[94] Woodward SH Friedman MJ Bliwise DL. Sleep and depression in combat-related PTSD inpatients. Biol Psychiatry. (1996) 39:182–92. 10.1016/0006-3223(95)00104-2 8837979

[95] Werner KB Griffin MG Galovski TE. Objective and subjective measurement of sleep disturbance in female trauma survivors with posttraumatic stress disorder. Psychiatry Res. (2016) 240:234–40. 10.1016/j.psychres.2016.04.039 27124208 PMC4885771

[96] Gehrman PR Harb GC Cook JM Barilla H Ross RJ. Sleep diaries of Vietnam War veterans with chronic PTSD: the relationships among insomnia symptoms, psychosocial stress, and nightmares. Behav Sleep Med. (2015) 13:255–64. 10.1080/15402002.2014.880344 24617942

[97] Ronzitti S Loree AM Potenza MN Decker SE Wilson SM Abel EA. Gender differences in suicide and self-directed violence risk among veterans with post-traumatic stress and substance use disorders. Women's Health Issues. (2019) 29(Suppl. 1):S94–102. 10.1016/j.whi.2019.04.010 31253249

[98] LeBouthillier DM McMillan KA Thibodeau MA Asmundson Gordon JG Types and number of traumas associated with suicidal ideation and suicide attempts in PTSD: findings from a US Nationally representative sample J Traumatic Stress. (2015) 28:183–90. 10.1002/jts.22010 25990916

[99] Eaton KM Messer SC Garvey Wilson AL Hoge CW. Strengthening the validity of population-based suicide rate comparisons: an illustration using US military and civilian data. Suicide Life Threat Behav. (2006) 36:182–91. 10.1521/suli.2006.36.2.182 16704323

[100] Rothberg JM Bartone PT Holloway HC Marlowe DH. Life and death in the, U. S, army in corpore sano JAMA. (1990) 264:2241–4. 10.1001/jama.1990.03450170089028 2214102

[101] Kuehn BM. Soldier suicide rates continue to rise: military, scientists work to stem the tide. JAMA. (2009) 301:1111–3. 10.1001/jama.2009.342 19293405

[102] Hoge CW Castro CA. Preventing suicides in, U. S, service members and veterans: concerns after a decade of war. JAMA. (2012) 308:671–2. 10.1001/jama.2012.9955 22893160

[103] Conner KR Bohnert AS McCarthy JF Valenstein M Bossarte R Ignacio R. Mental disorder comorbidity and suicide among 2.96 million men receiving care in the Veterans Health Administration health system. J. Abnorm. Psychol. (2013) 122:256–63. 10.1037/a0030163 23088376 PMC8372244

[104] Hyman J Ireland R Frost L Cottrell L. Suicide incidence and risk factors in an active duty, U. S, military population. Am J Public Health. (2012) 102(Suppl. 1):S138–46. 10.2105/AJPH.2011.300484 22390588 PMC3496445

[105] Ilgen MA Bohnert ASB Ignacio RV McCarthy JF Valenstein MM Kim HM. Psychiatric diagnoses and risk of suicide in veterans. Arch Gen Psychiatry. (2010) 67:1152–8. 10.1001/archgenpsychiatry.2010.129 21041616

[106] LeardMann CA Powell TM Smith TC Bell MR Smith B Boyko EJ. Risk factors associated with suicide in current and former, U. S, military personnel. JAMA. (2013) 310:496–506. 10.1001/jama.2013.65164 23925620

[107] Bullman T Schneiderman A Gradus JL. Relative importance of posttraumatic stress disorder and depression in predicting risk of suicide among a cohort of vietnam veterans. Suicide Life Threat Behav. (2019) 49:838–45. 10.1111/sltb.12482 29926933

[108] Conner KR Bossarte RM He H Arora J Lu N Tu XM. Posttraumatic stress disorder and suicide in 5.9 million individuals receiving care in the veterans health administration health system. J Affect Disord. (2014) 166:1–5. 10.1016/j.jad.2014.04.067 25012403

[109] Shen YC Cunha JM Williams TV. Time-varying associations of suicide with deployments, mental health conditions, and stressful life events among current and former, US military personnel: a retrospective multivariate analysis. Lancet Psychiatry. (2016) 3:1039–48. 10.1016/S2215-0366(16)30304-2 27697514

[110] Zivin K Kim HM McCarthy JF Austin KL Hoggatt KJ Walters H. Suicide mortality among individuals receiving treatment for depression in the Veterans Affairs health system: associations with patient and treatment setting characteristics. Am J Public Health. (2007) 97:2193–8. 10.2105/AJPH.2007.115477 17971541 PMC2089109

[111] Gradus JL. Posttraumatic stress disorder and death from suicide. Curr Psychiatry Rep. (2018) 20:98. 10.1007/s11920-018-0965-0 30221328

[112] Bishop TM Britton PC Knox KL Pigeon WR. Cognitive behavioral therapy for insomnia and imagery rehearsal in combat veterans with comorbid posttraumatic stress: a case series. Mil Behav Health. (2016) 4:58–64. 10.1080/21635781.2015.1100564 27695657 PMC5042213

